# A gradient of hemisphere-specific dorsal to ventral processing routes in parieto-premotor networks

**DOI:** 10.1162/netn_a_00407

**Published:** 2024-12-10

**Authors:** Marvin Jüchtern, Usman Jawed Shaikh, Svenja Caspers, Ferdinand Binkofski

**Affiliations:** Department of Clinical Cognition Science, Clinic of Neurology at the RWTH Aachen University Faculty of Medicine, ZBMT, Aachen, Germany; Institute for Neuroscience and Medicine (INM-1), Research Centre Jülich GmbH, Jülich, Germany; Institute for Anatomy I, Medical Faculty & University Hospital Düsseldorf, Heinrich-Heine-Universität Düsseldorf, Düsseldorf, Germany; JARA-BRAIN, Juelich-Aachen Research Alliance, Juelich, Germany; Institute for Neuroscience and Medicine (INM-4), Research Center Jülich GmbH, Jülich, Germany

**Keywords:** Dorsal stream, Frontoparietal, Graph theory analysis, Structural connectivity, Tractography

## Abstract

Networks in the parietal and premotor cortices enable essential human abilities regarding motor processing, including attention and tool use. Even though our knowledge on its topography has steadily increased, a detailed picture of hemisphere-specific integrating pathways is still lacking. With the help of multishell diffusion magnetic resonance imaging, probabilistic tractography, and the Graph Theory Analysis, we investigated connectivity patterns between frontal premotor and posterior parietal brain areas in healthy individuals. With a two-stage node characterization approach, we defined the network role of precisely mapped cortical regions from the Julich-Brain atlas. We found evidence for a third, left-sided, medio-dorsal subpathway in a successively graded dorsal stream, referencing more specialized motor processing on the left. Supplementary motor areas had a strongly lateralized connectivity to either left dorsal or right ventral parietal domains, representing an action-attention dichotomy between hemispheres. The left sulcal parietal regions primarily coupled with areas 44 and 45, mirrored by the inferior frontal junction (IFJ) on the right, a structural lateralization we termed as “Broca’s-IFJ switch.” We were able to deepen knowledge on gyral and sulcal pathways as well as domain-specific contributions in parieto-premotor networks. Our study sheds new light on the complex lateralization of cortical routes for motor activity in the human brain.

## Author Summary

Human motor abilities are processed via specialized yet intertwined pathways in the parietal and premotor cortex. These can be parcellated into networks of brain areas, sharing connection patterns that differ between hemispheres. We differentiated a set of graded pathways, connecting definable network hubs. The well-known Dorsal Stream for visuomotor transformation appears to be left-dominantly divisible into three distinct substreams, providing more anatomical detail about its specialization into visual and semantic segments. Supplementary motor areas show lateralized couplings with left dorsal and right ventral parietal areas, respectively, while left-sided AIPS connectivity to area 44/45 is mirrored by right-sided intersulcal links to the inferior frontal sulcus, both deepening our understanding of incorporated multi-task faculties like attention and speech.

## INTRODUCTION

Brain regions in the human frontal and parietal cortices process abilities that allow us to interact with our environment, arranged in functionally specific and structurally overlapping networks.

These abilities include skills like praxis, visuomotor transformation (Jeannerod et al., 1995), attention (Corbetta & Shulman, 2002; Thiebaut de Schotten, Dell’Acqua, et al., 2011), working memory, language (Saur et al., 2008), and tool use (Orban & Caruana, 2014), particularly serving the realization of target-directed movements (Sakreida et al., 2016; Verhagen et al., 2013). Basic principles for segregated circuits of parieto-premotor communication were first established through histological tracing and electrical stimulation in nonhuman primate brains (Rizzolatti & Luppino, 2001; Rizzolatti et al., 1998). It was proven that information travels between specialized, often complementary parietal and frontal areas, for instance, the ventral and dorsal stream (Rizzolatti & Matelli, 2003). The essential idea was that the posterior parietal cortex (PPC) receives input for different sensory modalities. Depending on the activation onset and intensity, specific projections dominate outgoing signals to, among others, premotor domains (Rizzolatti & Luppino, 2001; Rizzolatti et al., 1998). Hence, areas in the PPC and premotor cortex (PMC) function as places of integration and transformation, allowing for stimulus-adjusted action.

With the help of structural and functional brain imaging, individual cortical areas have been associated with distinct human abilities. In the current study, we were focusing on parietal and premotor association brain areas, which form a key network for movement processing and its supportive faculties.

Broca’s area (area 44, area 45) in the ventrolateral prefrontal cortex (VLPFC) is well-known for its role in language (Saur et al., 2008), which is realized through a complex network of mainly left lateralized brain regions, like the adjacent inferior frontal sulcus (IFS) (Bradler, 2015) and the inferior frontal junction (IFJ) (Friederici & Gierhan, 2013). Furthermore, areas 44 and 45 are associated with object identity (Rottschy et al., 2012), movement analysis (Binkofski et al., 2000), and motor programs (Ramayya et al., 2010). While the IFS is also responsible for working memory and cognitive tasks (Bradler, 2015; Van Doren et al., 2010), the more caudal IFJ appears important for attention (Zanto et al., 2010) and cognitive control (Friederici & Gierhan, 2013).

The core PMC in humans comprises a ventral premotor cortex (PMv), usually associated with working memory (Liu & Pleskac, 2011) and motor planning (Nuttall et al., 2018), especially for grasping movements (Tomassini et al., 2007), as well as a dorsal portion (PMd), known to be engaged in cognition (Genon et al., 2017), reaching, and hand movements (Caspers et al., 2010).

Most dorsally, the supplementary motor area (SMA) is composed of an SMAproper and a PreSMA subregion, dealing with language (through ventral connectivity) (Hertrich et al., 2016), motor learning, planning and execution (Genon et al., 2017), as well as action timing (Passingham & Lau, 2019).

The PPC can be divided into an inferior and superior parietal lobule (IPL, SPL), separated by an anterior and posterior portion of the intraparietal sulcus (aIPS, pIPS). It is important to mention that the terms “AIP” and “PIP” are also used for more distinct subareas of the intraparietal sulcus (IPS) in macaque monkeys (Orban et al., 2006) and should not be confused with our current division of the human IPS into an anterior (aIPS) and a posterior (pIPS) segment.

While most of the IPL is active during attention tasks (Binkofski, Klann, & Caspers, 2016; Caspers et al., 2011; Corbetta & Shulman, 2002), language (Binkofski et al., 2016; Caspers et al., 2013), motor preparation, and planning of gestures, often for tool use (Caspers et al., 2011; Ramayya et al., 2010), especially areas in the supramarginal gyrus are putatively relevant for the mirror neuron system (Rizzolatti & Craighero, 2004) and coding of near-body objects (Brozzoli et al., 2011).

Both the aIPS and the pIPS are involved in attentional processes (Gillebert et al., 2013), while the former also shares information for reaching (Rodríguez-Herreros et al., 2015) and the latter is rather known for its involvement in arm/eye movement control (Konen et al., 2013) and language skills (Richter et al., 2019). Similarly, superior parietal hubs got associated with the processing of attention (Roski et al., 2013) and reaching movements (Diedrichsen et al., 2005).

It is important to note that many of these faculties are highly lateralized in the human brain, such as praxis in the left hemisphere (Jeannerod et al., 1995), musical processing in the right IFS (Bradler, 2015), word listening in the left IPL, and nonlinguistic listening in the right SMA (Hesling et al., 2019). Since hemispheric lateralization plays only a marginal role in the nonhuman primate cortex (Passingham, 2008), it becomes evident that segregated and graded cortico-cortical pathways must serve important purposes for complex, multitask behavior in humans.

Another key feature of the human-environment interaction is the visuomotor transformation for object interaction. It is known to be processed along integrated, but separate, dorsal substreams, that is, a dorso-dorsal and ventro-dorsal pathway (Binkofski & Buxbaum, 2013; Rizzolatti & Matelli, 2003), tracing back to the two-stream model of visual processing in nonhuman primates (Mishkin & Ungerleider, 1982) and humans (Goodale & Milner, 1992), which was very recently extended by a third, lateral pathway, involved mainly in social cognition aspects of visual processing (Pitcher & Ungerleider, 2021). While online action control happens mainly dorso-dorsally along the SPL and dorsal premotor domains, the ventro-dorsal stream (including aIPS, IPL, and PMv) serves more semantic aspects of space perception and action understanding (Binkofski & Buxbaum, 2013; Rizzolatti & Matelli, 2003). These features enable humans not only to use tools in a proper manner but also to cognitively process interaction and consciously observe the behavior of others (Orban & Caruana, 2014). For such multilayered processing of sensory perception, conceiving, and (re)active output coordination, integrated cortical circuits are necessary.

Consequently, in the current study, we aimed at getting a deeper understanding of connectivity lateralization and network contribution of posterior parietal and premotor brain areas. We therefore used the recently proposed, finely parcellated regions of interest (ROIs) from the Julich-Brain atlas (Amunts et al., 2020) and applied a de novo two-stage procedure for characterizing these areas as nodes in the respective networks. High-resolution diffusion-weighted magnetic resonance imaging (DW-MRI), probabilistic tractography, and Graph Theory Analysis (GTA) allowed us to depict the shortest paths of parieto-premotor couplings, located on segments of larger interlobar routes. We expected regionally specialized cortical circuitries, including functionally relevant differences between the two hemispheres and between segments of network pathways, representing the complexity of human multitask processing.

## MATERIALS AND METHODS

### Subjects

The current study included 40 right-handed healthy adults (21 females and 19 males, aged 20 to 62 years, median: 23.5 years, average: 25.0 years, standard deviation (*SD*): 7.0 years) who were recruited through advertising on campus. All subjects had no history of neurological disease and brain or heart surgery. Informed consent was obtained from each participant before the experiment. The study protocol was approved by the RWTH Aachen University Independent Ethics Committee (EK 077/16). All experiments were performed in accordance with the guidelines of the Declaration of Helsinki.

### MRI Acquisition

All scans were performed on a Siemens MAGNETOM Prisma 3 Tesla MRI scanner (Siemens Medical Systems, Erlangen, Germany). Participants were instructed to lie calmly and move as little as possible during the measurement. A 20-channel head coil was used. High-resolution T_1_-weighted images were obtained by magnetization prepared rapid-acquisition gradient echo sequences with parameters as follows: 1.0-mm slice thickness with no interslice gap, 192 slices, time repetition (TR) = 1,900 ms, echo time (TE) = 2.21 ms, time to inversion = 900 ms, number of excitations (NEX) = 1, in-plane acquisition matrix = 256 × 256, time of acquisition (TA) = 4:18 min.

Diffusion tensor images were acquired using a single-shot Echo-planar imaging-based sequence: whole-brain coverage, 1.5-mm slice thickness with no interslice gap, 92 axial slices, TR = 3,230 ms, TE = 89.2 ms, NEX = 1, in-plane acquisition matrix = 140 × 140 with 75% phase partial Fourier, FOV = 210 × 210 mm^2^, TA = 5:41 min. We applied 99 diffusion directions with b-values = 0, 1,500 and 3,000 s/mm^2^ in terms of a multishell acquisition scheme to benefit from a stronger MRI signal of a lower b-value as much as higher angular contrast of a larger b-value to obtain more accurate local fiber estimations (Jbabdi et al., 2012).

### Preprocessing and Tractography

All preprocessing steps were executed in FSL (FMRIB’s Software Library, version 6.0, URL: https://fsl.fmrib.ox.ac.uk/fsl/fslwiki). To correct for susceptibility-induced distortions, we applied the topup function (Andersson et al., 2003; Smith et al., 2004). Nonbrain tissue was removed via the BET tool (Smith, 2002). Distortion correction of eddy currents and subject head motion was conducted using the eddy tool (Andersson & Sotiropoulos, 2016).

For registration, the respective T1-weighted image was co-registered to the corresponding b = 0 image in the diffusion MRI space, using the FLIRT tool (Jenkinson et al., 2002; Jenkinson & Smith, 2001). On the newly created T1/b0 image, an inverse transformation was applied to warp the Julich-Brain atlas masks from the Montreal Neurological Institute (MNI) space to the diffusion MRI native space. The ROIs in this atlas were created probabilistically from a set of postmortem brain samples, merged into a whole-brain template, where, for each voxel, the probability of all cytoarchitectonic brain areas was considered to determine the most probable assignment (Eickhoff et al., 2005).

On the preprocessed diffusion data, we applied the BedpostX function (Hernández et al., 2013), using a multishell ball and zeppelins deconvolution (Sotiropoulos et al., 2016) as well as a Rician noise model instead of default Gaussian noise (Jbabdi et al., 2012). The output was used in probabilistic tractography via Probtrackx (Behrens et al., 2007).

In Probtrackx, we used a multiple-mask approach with 5,000 individual samples (i.e., streamlines) drawn from the center of each voxel. A symmetric ROI-by-ROI connectivity matrix was generated for all 37 brain areas, where each cell contains the probabilistic number of tracks seeded from a certain ROI, reaching another ROI. Anonymized connectivity matrices for all participants can be found as shared data in an open repository (DOI: 10.6084/m9.figshare.21814770).

The anatomical location of the network’s ROIs can be found in Figures 1.1 and 1.2 in the MNI space. Please note that a depiction of the right hemisphere network was omitted due to its highly similar topography to avoid redundancy.

**Figure 1.**
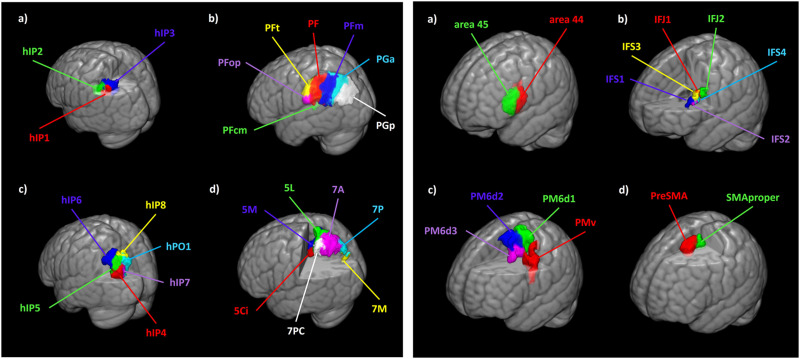
Localization of parietal ROIs in MNI space, left hemisphere. 1.1. Left-sided parietal ROIs on a three-dimensional brain template in MNI space. (A) aIPS: hIP1 (red), hIP2 (green), and hIP3 (blue). (B) IPL: PF (red), PFcm (green), PFm (blue), PFop (purple), PFt (yellow), PGa (light blue), and PGp (white). (C) pIPS: hIP4 (red), hIP5 (green), hIP6 (blue), hIP7 (purple), hIP8 (yellow), and hPO1 (light blue). (D) SPL: 5Ci (red), 5L (green), 5M (blue), 7A (purple), 7M (yellow), 7P (light blue), and 7PC (white). Localization of premotor ROIs in MNI space, left hemisphere. 1.2. Left-sided premotor ROIs on a three-dimensional brain template in MNI space. (A) VLPFC: area 44 (red) and area 45 (green). (B) IFS: IFJ1 (red), IFJ2 (green), IFS1 (blue), IFS2 (purple), IFS3 (yellow), and IFS4 (light blue). (C) PMC: PMv (red), PM6d1 (green), PM6d2 (blue), and PM6d3 (purple). (D) SMA: PreSMA (red) and SMAproper (green).

Please also find the comprehensive overview on the network’s ROIs in the Supporting Information: A schematic overview on the network’s anatomy is given as Supporting Information Figure S1. Centers of gravity for bilateral ROIs are presented in Supporting Information Table S1; size and corresponding cortical areas in nonhuman primates for the network’s ROIs can be found in Supporting Information Table S2.

Prior to registration, the PMv ROI was manually created based on previous parcellation schemes for the human ventral PMC (cf., e.g., Callan et al., 2010). The left PMv had a centroid of −45.91, −5.64, 42.34 (*x*, *y*, *z*) and right PMv of 42.34, −5.70, 43.97.

### Postprocessing

Normalization of matrices was realized by division through the total number of generated tracts from a given seed in each subject (the so-called “waytotal”), as well as averaging by taking the mean of every (directional) ROI pair (i.e., a fronto-parietal and a parieto-frontal orientation). Our graph can therefore be classified as weighted-undirected because it contains edge weights (the probabilistic streamline count per link) and does not differentiate between afferent and efferent paths. Since binarization can be considered as oversimplifying and the probability of molecular displacement along a vector is thought to be the same no matter the direction on the vector, this graph type is often recommended (Yeh et al., 2021). Note that only parieto-premotor couplings (i.e., 23 parietal × 24 premotor = 322 ROIs) were included in the final analysis, omitting intralobular, local connections, which would have distorted the fronto-parietal connectivity analysis.

GTA tools facilitate the quantification of network properties by applying summary metrics (Rubinov & Sporns, 2010). In general, ROIs are called nodes (or vertices), while the connections between them, that is, the streamlines, are called edges (or links) of the graph. We used tools provided by the Brain Connectivity Toolbox (BCT; URL: https://www.brain-connectivity-toolbox.net) (Rubinov & Sporns, 2010).

In probabilistic connectomics, thresholding is a common tool for reducing weak connection weights as putative false positives in a network. Although a universally ideal threshold does not exist, most approaches decide for applying a specific range of thresholds to increase specificity of tractography data (Yeh et al., 2021). This multithreshold approach usually suggests a lower cutoff above 0.01 (normalized and averaged streamline count), since it seems adequate to include smaller but supposedly relevant long-distance couplings (Tsai, 2018). We decided for a two-step strategy where we first averaged all edge weights into a three-dimensional whole-group matrix (mean(P_ij_) + 2 × *SD*(P_ij_), where *SD* is the standard deviation and P_ij_ is an edge of the graph), on which we secondly applied a threshold range between 0.01 and 0.1 in steps of 0.0025, resulting in 37 thresholded matrices per hemisphere (Cao et al., 2013; Tsai, 2018).

For validation of adequacy of the used threshold range, we exerted three goodness criteria proposed by Yun and colleagues (2020): (a) connectedness, (b) modularity, and (c) small-worldness. By that, we ensured that the selected thresholded graphs (a) only contain nodes that remain connected to other nodes in the network, (b) can be internally well divided into smaller subnetworks (i.e., modules), and (c) provide clear segregative and integrative features as a network.

Firstly, all thresholded graphs were screened for connectedness. Please find the respective MATLAB formula within the Supporting Information as Supplementary Methods (MATLAB, MathWorks, Version 2019b, URL: https://www.mathworks.com/products/matlab.html). An undirected graph is connected if every pair of nodes is linked by a path (of one or more edges).

Secondly, the modularity of all graphs was examined using Newman’s spectral reordering algorithm (Newman, 2006), which is defined as:

$$Q=\frac{1}{l}\sum_{i,j\in N}\left(a_{ij}-\frac{k_{i}k_{j}}{l}\right)\delta_{m_{i},m_{j}},$$

where *m*_*i*_ is a module containing node *i* and *δ*_*m*_*i*_,*m*_*j*__ = 1 if *m*_*i*_ = *m*_*j*_, otherwise 0. *A*_*ij*_ stands for an element of the adjacency matrix, which becomes 1 if there is an edge between nodes *i* and *j*; *l* represents the number of edges in the graph, and *k* is a node’s degree.

As indicated in the Yun study, thresholded matrices with a maximized modularity “Q” of above 0.3 were supposed to be included, so that a high level of topological clustering was ensured.

Thirdly, it was tested if all thresholded graphs had a small-world topology. In short, a network is small-world if it combines functionally specialized modules (high segregation) and a robust number of intermodular links (high integration), while it appears to be more clustered than random networks (Uehara et al., 2014). For evaluation of “small-worldness,” the clustering coefficient (CC) as well as the characteristic path length (CPL) were computed on native and null model networks for each threshold. The CC is given as follows (Watts & Strogatz, 1998):

$$C=\frac{1}{n}\sum_{i\in N}C_{i}=\frac{1}{n}\sum_{i\in N}\frac{2t_{i}}{k_{i}\left(k_{i}-1\right)},$$

with *C*_*i*_ as the CC of node *i* (*C*_*i*_ = 0 for *k*_*i*_ < 2). The CPL is given as follows (Watts & Strogatz, 1998):

$$L=\frac{1}{n}\sum_{i\in N}L_{i}\sum_{i\in N}\frac{\sum_{j\in N,j\neq i}d_{ij}}{n-1},$$

with *L*_*i*_ as the average distance between node *i* and the rest of nodes.

Ideally, the CC would have a value above 1, while the CPL is approximately 1 (Gong et al., 2009). In Humphries and Gurney (2008), small-worldness was stated as:

$$S=\frac{\frac{C}{C_{\mathrm{rand}}}}{\frac{L}{L_{\mathrm{rand}}}}\gg 1,$$

where *C* stands for the CC and *L* for the CPL.

Every matrix with a quotient above 1 was classified as small-world and included in further analysis (Uehara et al., 2014). The thresholded connectivity matrices for both hemispheres can be accessed via an open repository (DOI: 10.6084/m9.figshare.21378246).

We used the distance matrix function by Dijkstra’s algorithm, which contains lengths of shortest paths between all pairs of nodes, to find important couplings between ROIs across the network (Dijkstra, 1959). For connectivity analysis, an average of each ROI-to-ROI connection (link) across subjects was used (mean(P_ij_) + 2 × *SD*(P_ij_)). Focusing on the fronto-parietal connectivity, we counted all connection weights below the first quartile (25th percentile) as highly linked. This is because higher probabilistic streamline counts are equivalent to shorter path lengths between nodes, interpreted as high connectivity. Since length weights differ relatively little between neighboring brain regions, we added a second, more rigorous cutoff to detect strongest links below the 15th percentile. Note that thresholding had a marginal effect on distance matrix outcomes, so we refrained from comparing different threshold levels of distance matrices to each other, only using the threshold level with highest density in both hemispheres (i.e., 0.01). Distances matrices can be accessed via an open repository (DOI: 10.6084/m9.figshare.21814827).

### Statistics

To examine connectivity data for statistical robustness between hemispheres, we applied a two-tailed paired-sample *t* test on each normalized and averaged parieto-premotor ROI-to-ROI connection (*n* = 322) across all 40 subjects. Links between ROIs were regarded significantly different between hemispheres to a significance level of *α* = 0.05, corrected for multiple comparisons with the help of Benjamini-Hochberg’s false discovery rate (FDR) (Benjamini & Hochberg, 1995) to adjust for an elevated type I error rate, using an open-source calculation tool (Hemmerich, 2016). *p* values for all tracts can be found as Table 1 in the Results section.

**Table 1.**
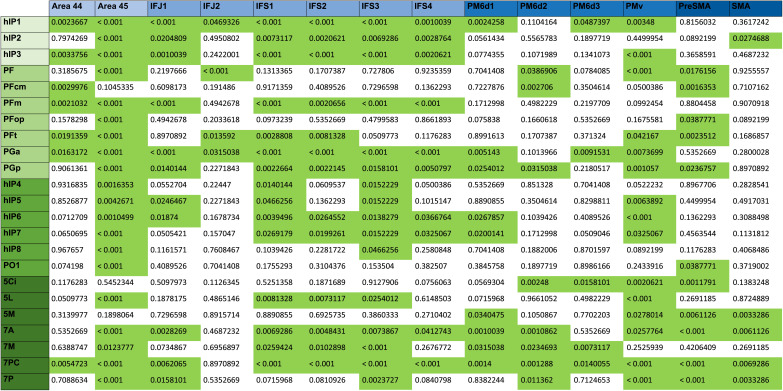
FDR-corrected *p* values for two-sided paired-sample *t* tests of connections between hemispheres

*N* = 322 two-sided paired-sample *t* tests, adjusted for elevated type I errors by FDR correction with a significance level of *α* = 0.05. *p* values for tracts between frontal (columns) and parietal (rows) ROIs across all 40 participants. Cells with light green background have a *p* value greater than 0.05. *p* values lower than 0.001 are written “<0.001” due to computational reasons.

Furthermore, to add information on the variability of the obtained data, we calculated the coefficient of variation (CV), which is the *SD* divided by the mean, for each connection (*n* = 322) in the two hemispheres across participants. To determine tracts with similar variability in the network, we applied a quartile clustering to the *SD* matrices of both hemispheres. The lowest *SD*s, below the first quartile of CV values in the hemisphere, were labeled as cluster A. Clusters B and C summarized tracts with an CV value below the second and third quartile, respectively. The highest CV values were grouped in cluster D. Complete CV matrices are obtainable as Supporting Information Figures S2.1 and S2.2.

### Node Characterization

Regarding network nodes, we propose a novel design for hub identification and characterization. It is based on principles of node centrality, while a “hub” is defined as a node with high centrality whatsoever. Centrality is the property of a node to interact with other nodes and thereby shape a network’s integration (Rubinov & Sporns, 2010). We usually differentiate individual aspects of centrality, such as degree (centrality), which describes the amount of neighboring links a node has (again distinguished between within-module and between-module connectivity), and betweenness centrality, which quantifies how often a node is located on a shortest path between a pair of nodes in the network (Rubinov & Sporns, 2010).

**Figure 2.**
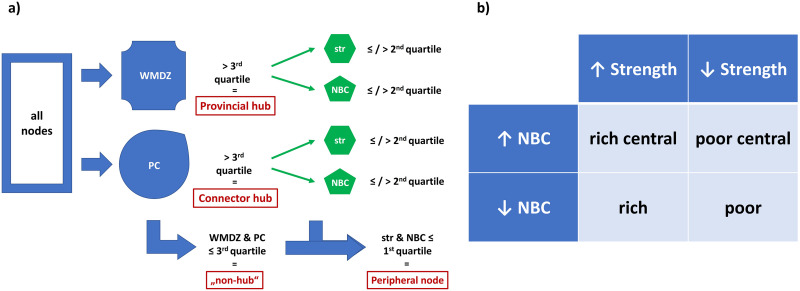
Node character assignment. (A) Every node was tested for hub status and type using WMDZ and PC. Hubs were further subclassified by strength (str) and NBC, while nonhubs were either called peripheral or remained unassigned. (B) Hub characterization depending on strength and NBC; “↑” means > second quartile, “↓” means ≤ second quartile.

We suggest a two-stage classification approach to define the specific role of brain regions in a network. For a schematic visualization, see Figure 2; for a detailed discussion of our approach and its interpretation, see the Supplementary Discussion at the Supporting Information. At the first stage, we used two functions to define network hubs: participation coefficient (PC) and within-module degree z-score (WMDZ).

The PC quantifies the portion of intermodular connectivity for a given node by using the community affiliation vector from a modularity function (Guimerà & Nunes Amaral, 2005). Since it displays the distribution of node connections among different modules, higher values of PC define “connector hubs” (or “intercluster hubs”), serving as bridges between separate clusters of nodes.

An exact description of the PC is given as follows (Guimerà & Nunes Amaral, 2005):

$$y_{i}=1-\sum_{m\in M}{\left(\frac{k_{i}\left(m\right)}{k_{i}}\right)}^{2},$$

defining *M* as the set of modules (provided by modularity) and *k*_*i*_(*m*) as the number of links between *i* and all nodes in module *m*.

The WMDZ is the within-module version of degree centrality in a given network and is typically associated with “provincial hubs” (or “intracluster hubs”) (Cohen & D’Esposito, 2016; Rubinov & Sporns, 2010)) that link vertices within a single cluster. In the same article, we find the mathematical definition of the WMDZ (Guimerà & Nunes Amaral, 2005):

$$z_{i}=\frac{k_{i}\left(m_{i}\right)-\bar{k}\left(m_{i}\right)}{\sigma^{k\left(m_{i}\right)}},$$

with *m*_*i*_ as a module containing node *I*, *k*_*i*_(*m*_*i*_) as the within-module degree of *i*, $\bar{k}$(*m*_*i*_) as the mean, and *σ*^*k*(*m*_*i*_)^ as the *SD* of the within-module *m*_*i*_ degree distribution.

By means of these two functions, we were able to classify nodes as either connector hubs (PC > third quartile in at least the highest and lowest threshold of all networks), provincial hubs (WMDZ > third quartile in at least the highest and lowest threshold of all networks), or nonhubs, with the latter failing both criteria. We did not include hubs in the final classification that lay above the PC/WMDZ cutoff only in occasional threshold graphs.

On the second stage, identified hubs were further differentiated and characterized by their degree and betweenness centrality, using two more graph theoretical tools from the BCT.

In weighted networks, the degree of a node is equivalent to its strength, which is the sum of weights of all edges linked to this node and highly proportional to its size (Farahani et al., 2019). Nodes with high strength (and relatively low betweenness) can be related to dense local connectivity, having rich input from the surrounding brain regions.

Node betweenness centrality (NBC) yields the fraction of all shortest paths in the network that contain a certain node, using the Brandes’ algorithm on connection-length matrices (Brandes, 2001). Freeman (1979) described the betweenness function in detail:

$$b_{i}=\frac{1}{\left(n-1\right)\left(n-2\right)}\sum_{\begin{array}{c} h,j\in N\\ h\neq j,h\neq i,j\neq i \end{array}}\frac{\rho_{hj}\left(i\right)}{\rho_{hj}},$$

where *ρ*_*hj*_ is the number of shortest paths between *h* and *j*, and *ρ*_*hj*_(*i*) is the number of shortest paths between *h* and *j* passing through *i*.

A high NBC means that a node shares many shortest paths in a network and is therefore in a topographically strategic position, either locally or between separate modules.

As a result, we computed strength and NBC for all nodes formerly defined as hubs by PC or WMDZ and prefixed them with a more concise nodal property, summarized in Figure 2. NBC matrices and strength tables can be found in an open repository (DOI: 10.6084/m9.figshare.21814902; DOI: 10.6084/m9.figshare.21814917). Additionally, nonhubs with strength and NBC values below the first quartile in the given network were called “peripheral nodes” (also cf. Guimerà & Nunes Amaral, 2005; Meunier et al., 2009); the remaining nodes went unclassified.

## RESULTS

### Left Hemisphere Connectivity

Using a cytoarchitectonic parcellation from the Julich-Brain atlas, we were able to describe connecting paths between 37 ROIs in fronto-parietal networks. Results from probabilistic tractography were interpreted with the help of GTA. For quantitative analysis of tractography outcomes, we applied Dijkstra’s distance matrix to the normalized and averaged connectivity matrices, including only parieto-premotor connections (i.e., 23 parietal × 14 premotor = 322 ROIs), representing shortest paths of streamlines passing through pairs of ROIs; for detailed results, see Tables 2 and 3. Paired-sample *t* tests (*α* = 0.05) were conducted on all ROI-to-ROI connections between hemispheres with *p* values FDR-corrected to control for statistical significance.

**Table 2.**
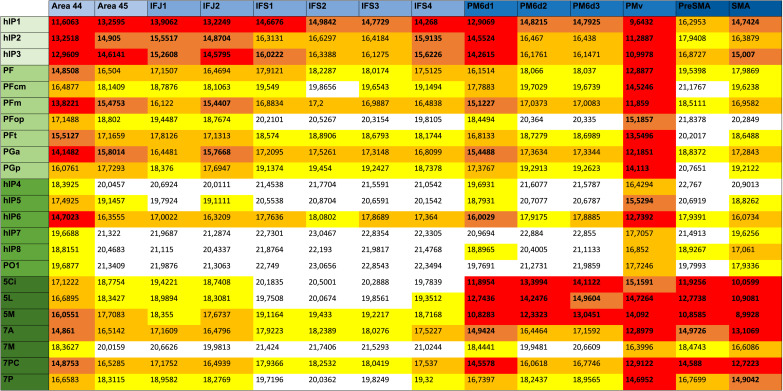
Distance matrix of left-sided fronto-parietal connectivity

Shortest weighted path matrix (D) by Dijkstra’s algorithm. Normalized, undirected, parieto-premotor graph with highest density threshold (0.01) for the left hemisphere. Coloring indicates a value below a certain percentile in the network: Below 15th percentile (red), 25th percentile (dark orange), 50th percentile (light orange), and 75th percentile (yellow), respectively.

**Table 3.**
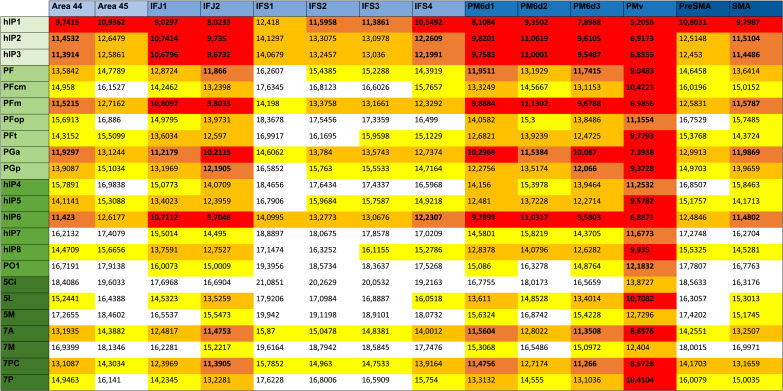
Distance matrix of right-sided fronto-parietal connectivity

Shortest weighted path matrix (D) by Dijkstra’s algorithm. Normalized, undirected, parieto-premotor graph with highest density threshold (0.01) for the right hemisphere. Coloring indicates a value below a certain percentile in the network: below 15th percentile (red), 25th percentile (dark orange), 50th percentile (light orange), and 75th percentile (yellow), respectively. Note that allover shortest paths were tendentially shorter than on the left.

The largest patterns of connected ROIs on the left include an aIPS-area 44-Premotor group as well as an SPL-Premotor-SMA group; while in the right hemisphere, the former changed to aIPS-IFJ-Premotor connectivity and the latter toward IPL-areas plus human Intraparietal Area 6 (hIP6) connected to IFJ and PMC.

In detail, inferior frontal areas were well-linked to all aIPS and some IPL areas. While area 44 showed most parietal connections, especially with hIP6, 5M, 7A, and 7PC, left area 45 and IFJ2 resembled each other in having strong connections to aIPS, PFm, and PGa. IFS areas as well as IFJ1 primarily linked to parts of the aIPS, which we called intersulcal connections, in contrast to gyro-sulcal connections, primarily between area 44/45 and IPL areas. Connections between area 44 and hIP1 (*p* = 0.002), hIP3 (*p* = 0.003), PFcm (*p* = 0.003), PFm (*p* = 0.002), PFt (*p* = 0.019), and PGa (*p* = 0.016) were significant between hemispheres, while this was true for most area 45 couplings except for three (PFcm, *p* = 0.105; 5Ci, *p* = 0.545; 5M, *p* = 0.190). IFS couplings were comparatively variable among subjects, with most tracts being among clusters C and D, especially to parietal sulcal areas. On the contrary, area 44 and 45 connectivity was less variable, including cluster A and B couplings with all parietal ROIs but hIP1.

PMv provided the overall shortest path lengths to parietal areas, connecting strongly to many IPL and posterior SPL areas and, together with PM6d1, to the whole aIPS (significant for PMv-hIP1, *p* = 0.003; PMv-hIP3, *p* ≤ 0.001; and PM6d1-hIP1, *p* = 0.002), with most couplings belonging to the least variable clusters A and B. For the PMC, PM6d3 links were the most variable, especially with IPS areas. Dorsal premotor and SMAs showed diverging linkage, compared with their inferior frontal counterparts, with strongest couplings to all anterior SPL nodes. PreSMA and, most notably, SMAproper were well associated with areas 7A (*p* ≤ 0.001, *p* = 0.006) and 7PC (*p* ≤ 0.001, *p* = 0.007), the former grouped in clusters A and B, denoting low variability among subjects.

From the perspective of the PPC, left aIPS mainly coupled with area 44/45, IFJ, and PMv, with hIP2 showing the least connectivity of the three subdomains. In the left IPL, PFm and PGa had especially strong connections with area 44/45 and PMv. In the left pIPS, hIP6 had the strongest connections to the frontal cortex, particularly again to area 44 and PMv, while the remaining ROIs poorly, and variably, connected to the frontal cortex. Finally, the left SPL areas showed a clear schism between anterior SPL, 7A and 7PC on the one hand and 7M plus 7P on the other, with the latter sparsely linking to frontal brain regions at all. 5Ci, 5L, and 5M highly linked to all of PMC and SMA; 7A had strong connections to PMv and SMAproper; and 7PC was linked with PM6d1, PMv, and both SMAs. Some of the most significant links (*p* ≤ 0.001) of the SPL between hemispheres were area 45 (with 5L, 7A, 7PC, and 7P), IFS1–4 (with 7PC), PMv (with 7PC and 7P), and PreSMA (with 7PC and 7P). Most SPL links showed low variability between subjects.

### Right Hemisphere Connectivity

Area 44/45 were remarkably less connected to the parietal cortex on the right than on the left, while IFJ1 and IFJ2 seemed to mirror area 44’s role in the left cortex by coupling intensely and significantly with aIPS (IFJ1-hIP1, *p* ≤ 0.001; IFJ2-hIP1, *p* = 0.047; IFJ1-hIP2, *p* = 0.020; IFJ1-hIP3, *p* = 0.001), PFm (with IFJ1, *p* ≤ 0.001), PGa (with IFJ1, *p* ≤ 0.001 and IFJ2, *p* = 0.032), and hIP6 (with IFJ1, *p* = 0.019). Least variability was found for areas 44 and 45, while sulco-sulcal connections had most variability, even though less for AIPS-IFS connections than on the left.

Core premotor areas heavily connected to the parietal cortex, with dorsal premotor areas also reaching PFm, PGa, and hIP6. Links between PM6d1 and PGa (*p* = 0.005), PM6d1 and hIP6 (*p* = 0.027), as well as PM6d3 and PGa (*p* = 0.009) were significantly lateralized. Compared with the left, especially PM6d2 had a larger share of cluster A and B connections especially with the IPL. Interestingly, both SMAproper and PreSMA lacked stronger couplings to any SPL area, in contrast to the left hemisphere, rather coupling with aIPS (SMAproper-hIP2, *p* = 0.027) and IPL (e.g., PreSMA-PFcm, *p* = 0.002; PreSMA-PFt, *p* = 0.002), nearly all of which fell under clusters A and B for a low CV.

The right aIPS provided strong connections to nearly all frontal ROIs, with hIP2 and hIP3 performing almost identically. From the IPL, similar to the left hemisphere, strongest couplings existed to PFm and PGa. In the right pIPS, only hIP6 had thorough connections with frontal areas, however, even stronger than on the left, notably to IFJ (significant to IFJ1, *p* = 0.019, but high variability) and all PMC areas (significant to PM6d1, *p* = 0.027, and PMv, *p* ≤ 0.001, cluster B and C variability). Only 5L strongly coupled with PMv and while 7M and 7P resembled the situation in the left cortex, 7A and 7PC switched connections from area 44 (on the left) to IFJ2 (significant for 7PC-area 44, *p* = 0.005). Notably, SPL connections to PMv were even stronger than on the left (insignificant only to 7M, *p* = 0.253, all but one in cluster A).

### Bilateral Node Characterization

After calculating the connectivity between frontal and parietal brain areas to describe *where* information is primarily transferred, we were interested in analyzing, *which* of the network hubs is responsible for local or long-distance exchange. The idea behind this was that the connectional fingerprint of a brain area can define its structural role in a network (Passingham et al., 2002).

Therefore, brain areas were classified into nine categories of nodes in the network using a two-stage evaluation process: We first checked for hub criteria, using the PC and the WMDZ for each brain area. Second, we analyzed the integrative features of the hub by measuring its “strength” and NBC. Please find a more detailed explanation of the used GTA tools and their interpretation in the Materials and Methods section as well as a further discussion in the Supporting Information (Supplementary Discussion).

In short, there were two types of hubs, based on nomenclature from the literature (Passingham et al., 2002; Rubinov & Sporns, 2010; Sporns et al., 2007): connector hubs, which are nodes that are important for (global) intermodular integration (Rubinov & Sporns, 2010) and have a PC above the third quartile of the network, and provincial hubs, facilitating modular segregation and local connectivity (Rubinov & Sporns, 2010) that are above the third quartile of nodes in WMDZ graphs.

These hubs were further assigned one of four categories to differentiate their exact role in the networks. We called a hub “rich” if the node strength (or “degree”) was among the top 50% of all nodes, “rich central” if NBC was additionally among the top 50% of nodes, “poor central” if only NBC was above the 50th percentile of all nodes, and “poor” if strength as well as NBC values lay below the 50th percentile. Central hubs lie on shortest paths (i.e., strong connections) between brain areas within (provincial hubs) or between modules (connector hubs) of a network. Please find a schematic visualization of hub roles in the network as Supporting Information Figure S3. The detailed hub assignment is given in Table 4.

**Table 4.** Bilateral node character assignment

**Left**	**Connector hub**	**Provincial hub**
**Rich central**	hIP3, PGa, hIP6, 7A	Area 45, hIP1, hIP2, PF, 5L
**Rich**	–	hPO1, IFS3
**Poor central**	PMv, PGp, 7PC, 7P	–
**Poor**	–	PM6d2
**Right**	**Connector hub**	**Provincial hub**
**Rich central**	PMv, hIP1, hIP3, PGp, hIP6, 7A	IFS4, PFm
**Rich**	–	Area 45, hIP5, hIP8
**Poor central**	7PC	PM6d1, PM6d2, 5L, 5M
**Poor**	PGa	–

Nodes in the left and right hemisphere were assigned a network role considering their hub properties using a two-stage characterization approach. Connector or provincial hubs were further specialized as rich central, rich, poor central, and poor, respectively.

All nodes that did not match PC and WMDZ criteria, called “nonhubs,” could be further specified as peripheral nodes, if both NDC and strength values were among the lowest (i.e., below the first quartile) in the networks (Zhang et al., 2021). We identified left PM6d3, right PreSMA, SMAproper, and PFcm as well as bilateral PFop, 5Ci, and 7M as peripheral nodes for having both strength and NBC values below the 25th percentile.

## DISCUSSION

In the current study, we used multishell DW-MRI, tractography, and GTA techniques to investigate the structural connectivity between concisely parcellated parietal and premotor brain areas in the human cortex. We did find not only distinct patterns of parieto-premotor pathway segments but also clear signs of lateralization between hemispheres.

### A Third, Lateralized Dorsal Substream?

Visuomotor transformation for tool use is known to be processed along two different, interconnected parts of the parieto-frontal dorsal stream: the dorso-dorsal and the ventro-dorsal pathway (Binkofski & Buxbaum, 2013; Rizzolatti & Matelli, 2003). In the present depiction of the dorsal stream, intra- and interhemispheric gradation of connectivity becomes evident. Parieto-frontal information might be processed in more than two dorsal substreams, differing in the two hemispheres. Please find a visual delineation in Figures 3.1 and 3.2. The BrainNet Viewer software (Version 1.7, URL: https://www.nitrc.org/projects/bnv/) was used for visualization (Xia et al., 2013).

**Figure 3.**
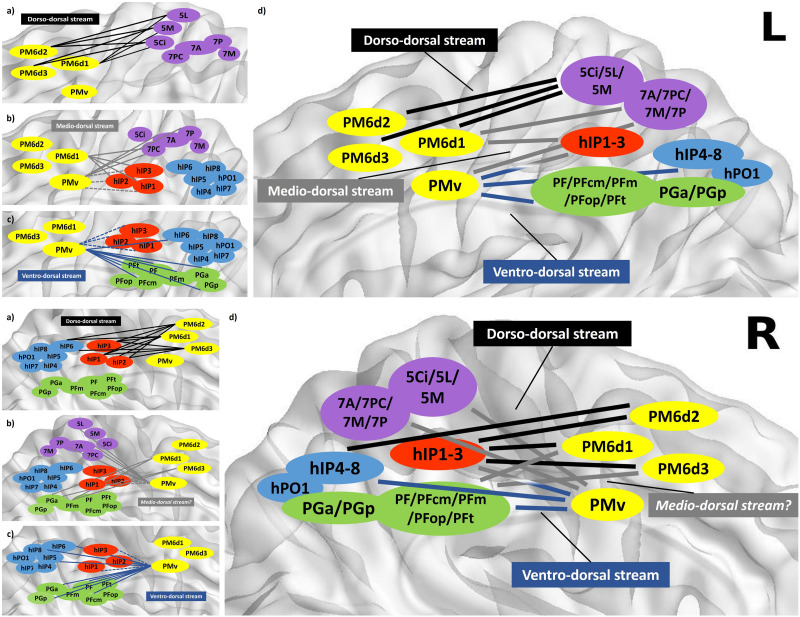
Outline of a lateralized dorsal stream, left hemisphere. 3.1. Depiction of strongest links (below the 15th percentile in the undirected distance matrix) between frontal and parietal ROIs in the left (L) hemisphere. (A–C) Detailed couplings of three overlapping substreams of the dorsal stream, comprising a dorso-dorsal (black), medio-dorsal (gray), and ventro-dorsal (blue) pathway. A schematic overview is given in (D). The two-colored (D) and dotted (B, C) hIP1–3/PMv arrows indicate ambiguous affiliation of the stream. Outline of a lateralized dorsal stream, right hemisphere. 3.2. Depiction of strongest links (below the 15th percentile in the undirected distance matrix) between frontal and parietal ROIs in the right (R) hemisphere. (A–C) Detailed connectivity in three overlapping substreams of the dorsal stream. A schematic overview is given in (D). A dorso-dorsal (black), medio-dorsal (gray), and ventro-dorsal (blue) pathway can be differentiated, putatively organized in a two- or threefold model, since a medio-dorsal pathway is less clearly delimitable than on the left. The two-colored (D) and dotted (B, C) hIP1–3/PMv arrows indicate ambiguous affiliation of the stream.

On the left, gradation and segregation appear very clearly, in that anterior SPL connectivity becomes gradually weaker from dorsal to ventral, while posterior SPL, pIPS, and IPL couplings behave the other way around. This was similarly seen in earlier functional connectivity studies, where superior dorsal PMC was primarily connected to the dorsal parietal domains and inferior PMd coupled strongly with intraparietal sulcal areas, while inferior parietal areas were well connected with the ventral PMC, presumably for grasping and tool use (Mars et al., 2011). Area aIPS performs in a slightly intermediate fashion, following an increasing dorso-ventral gradient as well, but without disconnecting from PMd in the case of hIP1. This coincides with hIP1’s role as a fronto-parietal connector hub and first hierarchy domain for goal-directed movements (Verhagen et al., 2013). Intriguingly, dorso-ventral gradation in the parietal cortex does not split patterns into a PMd and PMv pathway, but rather shows similar connectivity patterns of PM6d1 and PMv, drawing a fluent passage through the three dorsal premotor ROIs. This might be due to the more rostro-ventral position of PM6d1, but differences in the opposite hemisphere makes this purely topographical explanation questionable. Instead, we assume that the not-yet-subparcellated area PMv is further subdividable as well, putatively between a more ventral (“ventro-dorsal”) and a more dorsal (“medio-dorsal”) portion, the latter converging with PM6d1 in connective patterns. A threefold division of the parieto-frontal dorsal stream therefore propagates left-sided sensorimotor information on individual, but cross-linked processing pathways. Such interstream links were recently described for macaques (Greulich et al., 2020).

In our model, the dorso-dorsal path connects dorsal SPL with rostro-dorsal PMd, significant for 5Ci (*p* = 0.002, *p* = 0.016), 7M (*p* = 0.023, *p* = 0.007), and 7PC (*p* = 0.001, *p* = 0.014) to both PM6d2 and PM6d3, putatively for reaching (Diedrichsen et al., 2005; Rizzolatti & Matelli, 2003), hand movements (Caspers et al., 2010), and visuospatial imagery (Genon et al., 2017). These functions are also confirmed by the fact that lesions to the dorso-dorsal stream are known to cause optic ataxia (Karnath & Perenin, 2005). A medio-dorsal section couples ventral SPL and parts of the IPS with intermediate PMd/v, associated in the literature with movement coordination in space (Tomassini et al., 2007) and manual object manipulation (Binkofski, Buccino, Stephan, et al., 1999). Ventro-dorsal fibers would process grasping (Tomassini et al., 2007), target-oriented action (Sakreida et al., 2016), and decision-making skills (Caspers et al., 2008) via IPL, aIPS, pIPS, and ventral PMv. IPL-PMv couplings, especially, might transfer important semantic information for environment interaction, since lesion studies have shown that both PGa/PGp as well as PMv are important for reading, writing, and speech error perception (Binkofski et al., 2016; Nuttall et al., 2018), and ventro-dorsal stream pathology includes limb apraxia (Pisella et al., 2006).

On the right, two main differences become apparent. Firstly, the gradation of sulcal areas is less pronounced, especially for aIPS and hIP6, which show little preference between ventral and dorsal premotor areas. Differences between left and right were significant for PM6d1-hIP1 (*p* = 0.002), PM6d1-hIP6 (*p* = 0.027), and PMv to hIP1 (*p* = 0.003), hIP3 (*p* ≤ 0.001), and hIP6 (*p* ≤ 0.001). Additionally, SPL-dorsal premotor connectivity is much weaker, posing the question whether dorso-dorsal information propagates beyond the current network (e.g., to the dorso-lateral prefrontal cortex [dlPFC], for which there is good evidence; Caspers et al., 2012; Greulich et al., 2020; Jung et al., 2018; Uddin et al., 2010) or becomes part of a “medio-dorsal” PMv stream, corresponding to a presumably different internal PMv division.

This becomes more likely since, secondly, clear differences between connectivity fingerprints of dorsal premotor areas are absent in the right hemisphere, appearing much more as a unity than on the left, with PM6d1 and PM6d3 performing almost identically. A connective “boundary” appears more probable between right PMd and PMv, with the assumption of an internal splitting of upper and lower PMv. This is in accordance with not only more sulcal and inferior parietal links of right PMd, mostly significant for PM6d1 (e.g., PM6d1-hIP1, *p* = 0.002), but also for links between IPL and PM6d2 (e.g., with PFcm, *p* = 0.003) and PM6d3 (with PGa, *p* = 0.009), while PMv connectivity keeps mainly the same. As a consequence, visuomotor transformation on the right is either propagated via three slightly different streams (even though also of a ventral, medial, and dorsal design) or through the previously established dyad, fitting the model of a ventral and dorsal attentional system (Corbetta & Shulman, 2011), for instance. It is also important to note that a differentiation into substreams might occur earlier or later in their courses, similarly seen very recently for the human visual pathway between temporal and IPL areas (Choi et al., 2020).

As a limitation, one must mention that PM6d2 and PM6d3 links to parietal sulcal areas were mostly nonsignificant between hemispheres (see Table 1), together with a medium to high variability across subjects in PM6d3. This shows the need for verification of the current gyro-sulcal connections in a larger cohort.

This also casts new light on the well-established anatomical concept of three distinct superior longitudinal fasciculi (SLFs) (Thiebaut de Schotten, Dell’Acqua, et al., 2011). Dorsal SLF1, associated with voluntary orienting of spatial attention toward visual targets (Corbetta & Shulman, 2002), matches with parts of the left dorso-dorsal and SMA networks (see below) in the current study. However, its right counterpart appears to be part of a more ventral “medio-dorsal” stream. One explanation for its “reduced” dorso-dorsal connectivity on the right in our study could be that it is overlapping with the so-called dorsal visuospatial pathway, connecting occipitoparietal cortex and IPL with the dlPFC (Uddin et al., 2010), with the latter not being part of our current network and therefore missing in fiber track counts.

The middle SLF2 is thought to communicate between its dorsal and ventral neighbors as a modulator for dorsal networks (Thiebaut de Schotten, Dell’Acqua, et al., 2011), which can be compared with especially strong aIPS connectivity on the right and fits to our medio-dorsal substream on the left. Thiebaut de Schotten and colleagues described it as rather right-lateralized (Thiebaut de Schotten, Dell’Acqua, et al., 2011) so we assume that it is currently mirrored by stressed ventro-dorsal long-distance couplings, as between SPL and PMv, putatively forming a distinct medio-dorsal pathway on the right.

The ventral-most SLF3 is thought to get activated through automatic capture of spatial attention by visual targets (Corbetta & Shulman, 2002), probably overlapping with the arcuate fasciculus (AF) (Thiebaut de Schotten, Ffytche, et al., 2011). It is currently mirrored by IPS/IPL-PMv patterns in both hemispheres, seemingly the most stable in the network. Since PMv connectivity is complemented by even stronger SPL input on the right, such previously proposed lateralization (Thiebaut de Schotten, Dell’Acqua, et al., 2011) is confirmed by our data. However, its prefrontal aspects might be further accentuated by the “Broca’s-IFJ switch” (see below), with right-sided information predominantly reaching IFS areas, presumably for attention processing.

### A Functional Dichotomy of the SMA

The SMA is a highly sophisticated motor region of the human cortex that deals with motor learning, action sequencing, and rhythmicity (Genon et al., 2017).

The left-sided SPL connectivity of the SMA confirms its role in converting sensory input through gyro-gyral dorsal parietal pathways, forming a putative part of the dorso-dorsal stream. This would encompass areas 7PC and 7P, which were designated as poor-central connector hubs, therefore forming important domains for integration of the SPL.

**Figure 4.**
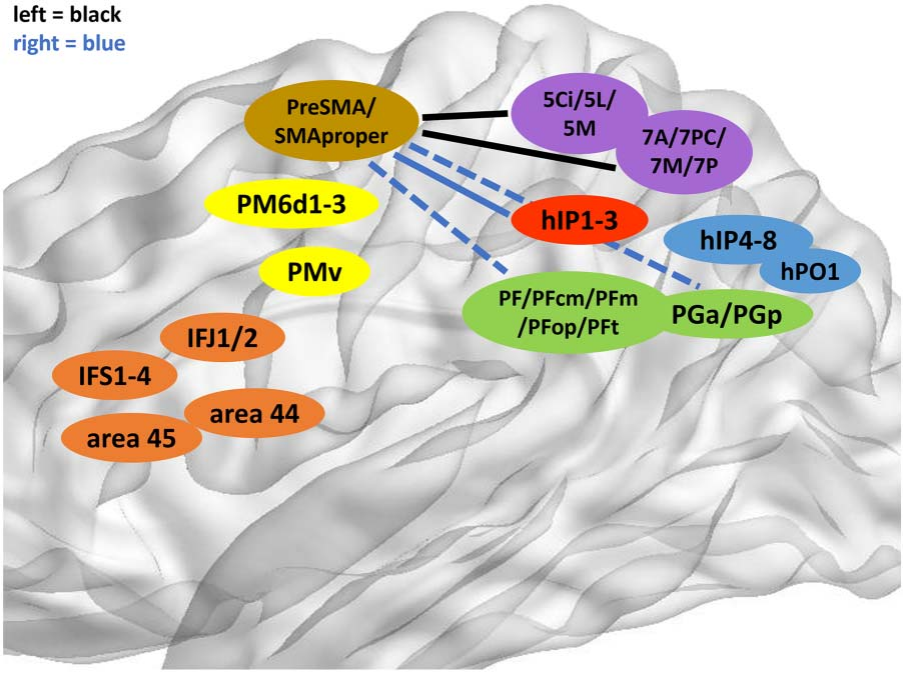
Outline of the SMA dichotomy. Depiction of strongest links between SMA and parietal ROIs on the left (black) and right (blue). For reasons of comparison, the left and right connectivities are both projected on the brain’s left hemisphere. A left dorso-dorsal pattern clearly differs from a right ventro-dorsal pattern. Continuous arrows indicate strongest connectivity (below the 15th percentile) in the undirected distance matrix, while dotted arrows stand for connectivity below the first quartile but above the 15th percentile.

Similar to right-sided SPL areas 5Ci, 5L, and 5M, the SMAs of the right hemisphere lack most of their dorsal parieto-frontal connections, significant especially for PreSMA (with 5Ci, *p* = 0.001 and 5M, *p* = 0.006). It is highly probable that such hemispheric divergence reflects functional segregation for multitask processing and might serve as an equivalent of proposed long-reach association fibers of the SPL (Caspers et al., 2012). Dorso-ventrally graded, right-sided gyro-sulcal and gyro-gyral couplings of the SMA with aIPS and IPL might, for instance, enable listening abilities, including attentional and executive processes (Hesling et al., 2019). A very interesting case of a patient with isolated gait apraxia after localized bilateral SMA infarction due to an anterior cerebral artery anomaly might underpin functionally relevant ventral parietal information flow to the right SMA (Della Sala et al., 2002). Especially, IPL shows strong connectivity with SMAproper in the right hemisphere (although insignificant, see Table 1), inspiring the idea that reciprocal SMA-IPL circuits could serve a more cognitive role in action understanding (Hertrich et al., 2016) and coordination of varying sensorimotor modalities (Nachev et al., 2008). On the other hand, considerably fewer parietal connections of the right SMA could reflect its putative right-lateralized integration into a cortico-subcortico-cerebellar motor network (Genon et al., 2017). A schematic overview of this SMA dichotomy can be examined in Figure 4.

As a sidenote, the fact that 7M had no strong frontal couplings whatsoever is presumably congruent with its role as a “connective bridge” to the temporal, rather than frontal lobe (Jung et al., 2017), similar to the occipito-parietal hub position of PGp (Caspers et al., 2013).

### The Broca’s-IFJ Switch

In the current data, connectivity between ventrolateral prefrontal and inferior parietal areas is substantially lateralized and segregated between two frontal domains. On the left, area 44 shows predominant gyro-sulcal and gyro-gyral connections with aIPS, hIP6, and IPL (especially PGa and PFm), for the most part significant (e.g., area 44-hIP1, *p* = 0.002), most probably involved in language processing (Binkofski et al., 2016; Caspers et al., 2011, 2013; Richter et al., 2019), hand movements, and object interaction (Binkofski, Buccino, Posse, et al., 1999). Right-sided IFS areas (especially IFJ) mirror this pattern (significant, e.g., IFJ1-hIP1, *p* ≤ 0.001), connoting a right-dominant intersulcal information flow between the IFS and the IPS, putatively serving attentional tasks (Caspers et al., 2011, 2012; Corbetta & Shulman, 2002) and working memory (Richter et al., 2019; Van Doren et al., 2010), which could therefore spread parallelly, enabling distinct multitask abilities. We call this a lateralized “Broca’s-IFJ switch” between motor-language (left) and attentional faculties (right).

When compared visually, the most striking difference between hemispheres are twofold streams connecting the aIPS and area 44/45 on the left, which represent the direct long segment and the indirect anterior segment (≈ SLF3) of the AF, with only the former substantially carrying fibers to IFJ. This is in accordance with the fact that the AF was shown to be left lateralized and therefore presumably more clearly presents its two main fronto-parietal branches in the left hemisphere (Thiebaut de Schotten, Ffytche, et al., 2011). On the right, aIPS-VLPFC routes seem to propagate more coherently in what resembles the third SLF. The hemispheric difference in connectivity could therefore also be mirrored in the difference of larger pathway architecture. A depiction of this can be found in Figures 5.1 and 5.2.

**Figure 5.**
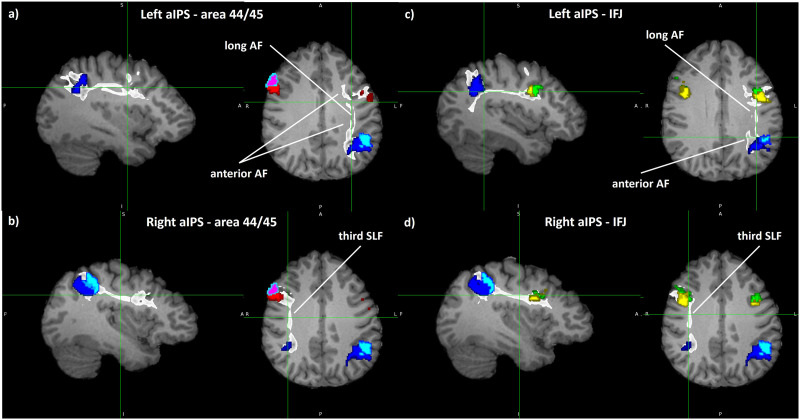
Depiction of bilateral tracts between aIPS, area 44/45, and IFJ. Exemplary single-subject visualization of connecting fiber tracts between the anterior intraparietal sulcus (aIPS) and area 44/45 (A, B) as well as the inferior frontal junction (IFJ) (C, D) in sagittal (left subimage) and axial (right subimage) planes. (A) The left-sided long (direct) and anterior segments of the AF connecting the aIPS and area 44/45 on the left. (B) The connecting third (ventral) part of the SLF between right-sided aIPS and area 55/45. (C) The left hemisphere course of both segments of the AF, with the long AF primarily connecting hIP1 and hIP2 with IFJ. The anterior AF does not substantially reach the IFJ but runs more dorso-medially. (D) Connections between the aIPS and IFJ via the third SLF in the right hemisphere. While two separate yet partially intertwined pathways form the connective routes on the left, a more uniform right sided pathway can be differentiated. Tracts (white) were generated probabilistically and bidirectionally, a brightness threshold between 10,000 and 15,000 as well as linear interpolation were used for illustration purposes in fsleyes (DOI: 10.5281/zenodo.7038115). Note that the software uses radiological space for template depiction, so left and right orientation is switched. The thin light green cross marks the position of focus in all three planes. Areas included: hIP1 (dark blue), hIP2 (light blue), area 44 (red), and area 45 (purple). A = anterior, P = posterior, L = left, R = right.

For both patterns, we find a dorsal to ventral gradation in connectivity strength. Notably, the “weaker” pattern of each hemisphere still solidly couples with most of aIPS, fittingly designated as rich-central connector hubs (left hIP3, right hIP1, and hIP3), along with hIP6 and PGa. Therefore, the “weaker” network part of each side would complement the main hemispheric function, that is, right-sided area 44 is involved in movement attention in space (Binkofski et al., 2000) and left-sided IFS areas are also activated by words and pictures (Van Doren et al., 2010). The right rich-central provincial hub PFm is in accordance with its proposed function as a (local) transition spot between the supramarginal and the angular gyrus (Binkofski et al., 2016).

Lastly, the functional relevance of the switch is obvious in that right-sided disruption of fibers connecting parietal and prefrontal areas lead to visuospatial neglect (Urbanski et al., 2008), while congruent left-sided lesions can cause aphasia (Madhavan et al., 2016) and apraxia (Culham & Valyear, 2006).

### Gyral and Sulcal Connectivity

It became evident that gyri and sulci have different connectivity properties and, therefore, functional roles in cortical networks (Jiang et al., 2018). While gyri mostly serve as interregional connection centers, sulci often represent local integration units (Deng et al., 2014).

Gyro-gyral connections were stated to represent the strongest functional pathways, transmitting information among remote brain regions. This could be confirmed by long-distance couplings between larger gyral ROIs in the premotor (e.g., PMv and area 44) and PPC (e.g., PFm, PGa, and 5M). Size and position can substantially influence (and possibly overestimate) the functional role assigned to an ROI in a network, which is discussed in more detail in the Methodical Limitations section.

Gyro-sulcal couplings are thought to have “moderate” functionality as links between intra- and interregional routes. This is especially interesting for aIPS areas, having a topographically exposed position close to the central sulcus, representing an intersection point for both core premotor and (right-sided) supplementary motor areas as well as areas 44 and 45 on the left. Hence, we would like to stimulate the idea that sulcal areas in strategic positions, like hIP1, for instance, can be of higher functional relevance for pathway integration as formerly thought. Intersulcal connections are known for forming indirect circuits via gyri for long-distance integration (Deng et al., 2014), often assigned a purely local network aspect. In the current study, links between aIPS and pIPS, hIP1 and hIP6 in particular, with IFS areas prove that long-distance intersulcal connections complement gyral information flow. These could form putative simultaneous processing pathways for parallel information flow in the cortex, seen for the “Broca’s-IFJ switch,” possibly as grouped clusters of smaller brain areas forming local (and probably also regional) network hubs.

### Methodical Limitations

Importantly, the current study has focused on a distinct set of brain areas, covering portions of association cortices in the frontal and parietal lobe involved in motor activity, tool use, space perception, and interaction. With the advantage of a more concise investigation of intranetwork connectivity comes the disadvantage of excluding several neighboring brain regions, especially in the frontal cortex, potentially incorporated in some of the depicted subnetworks. It is therefore reasonable to put the presented results into relation with future connectivity studies, exploring associated structural and functional cortical networks.

It is important to mention that advanced multishell Diffusion Weighted Imaging and tractography implicate methodical limitations. Most notably, it remains difficult to reconstruct intersecting, crossing, or bordering streamlines, whereas smallest white matter fibers are generally not identifiable at all (Tournier et al., 2011).

Biases of tractography usually include the following (Girard et al., 2020): (a) Algorithms tend to better reconstruct short, large, and straight pathways; (b) streamlines close to gray matter or cerebrospinal fluid voxels tend to be underrepresented, sometimes causing spurious or incomplete fibers; and (c) pathways on the gyral crown are preferably considered in outputs as opposed to those in gyral walls. The latter aspect appears especially crucial since we aimed at depicting concise connectivity patterns as well in gyri as in sulci, even between small brain areas. Apart from possible anatomical reasons, the technical causes for this bias comprise voxel size, a low curvature threshold, and whole-brain seeding (Schilling et al., 2018). To compensate for this, we used a high-resolution voxel design, refrained from whole-brain seeding and aimed at explicitly depicting smaller connections by subdividing the network and averaging results. Another example for this gyral bias is inherent to the approach of using the CV, which is especially sensitive to changes in smaller mean values close to 0 and is therefore less meaningful for connection strength values of sulcal ROIs, typically yielding smaller streamline counts.

Conversely, small ROIs can affect the reconstruction of network pathways, being irregularly shaped or having a prominent position on a gyrus, influencing the depicted course of a pathway. We tried to compensate for this circumstance by using groups of smaller ROIs rather than single ROIs to assign segments of pathways and by drawing on centroid positions of ROIs in pathway visualization.

Although inverting streamline weights to lengths is deemed a suitable measure for interpretation of connectivity strengths (Yeh et al., 2021), it is important to mention that short distances can occur due to topographic proximity, high-edge weights as well as many edges connecting two nodes. Thereby, length values can have differing reasons, not always identical with what we understand as a “linkage” in the sense of internodal communication.

Another limitation specific to the present study is the use of a manually created ROI PMv, which was defined in collaboration with a neuroanatomist Svenja Caspers (SC) based on its established cortical position. On the one hand, it remains more difficult to compare with the rest of ROIs since it was not commensurably defined in its shape and subparcellation. On the other hand, due to its relatively large volume, it tends to “absorb” streamlines from parietal seeds, having strong impact on cutoff selection and centrality measures. Therefore, a reevaluation of the current PMv connectivity is recommendable with the use of multimodal parcellation.

### Conclusions

The current study had three major results concerning structural connectivity between human parietal and premotor brain areas:

1. A triadic dorsal stream,
2. heavily lateralized SMA couplings, and
3. a “switch” in connectivity patterns of area 44/45 and the IFJ between hemispheres.

Although less prominent on the right, a gradient of interconnected, yet distinct, substreams for visual perception and object manipulation serves as the anatomical equivalent of a functional gradient that both the dorsal stream and the ventral stream represent. We know that the visual guidance of action is mainly processed via the dorsal stream (originally starting in the primary visual cortex), while the semantic and cognitive aspects are largely processed ventrally (Goodale & Milner, 1992). Aside from the already deciphered dorso-dorsal and ventro-dorsal substreams, a medio-dorsal pathway, emerging in the parietal cortex and connecting mainly SPL, aIPS, PMv, and PM6d1 (again, depending on the hemisphere), is mainly gyro-sulcal and could be involved in hand motor function and direct object manipulation (Binkofski, Buccino, Stephan, et al., 1999; Jeannerod et al., 1995). With new insights into the internal subdivision of the ventral PMC, we will hopefully learn more about the exact anatomic groundwork of this processing path.

The found that the “dichotomy” of supplementary motor connectivity with the PPC builds upon knowledge on hemisphere-specific motor processing. Dorsally oriented couplings on the left would be closely related to the neighboring dorso-dorsal stream fibers, transferring sensory information about animate objects in space. Its right counterpart would rather serve attentional and cognitive functions in object interaction, necessary for multitask movement processing.

Similarly, ventrolateral prefrontal connections of the aIPS primarily reach areas 44 and 45 on the left for linguistic aspects of motor activity, whereas right-sided couplings are largely intersulcal, communicating attentional information via the IFS. This finding is especially relevant for understanding human cortex functioning as a bilateral, simultaneous network system, considering that structural and functional lateralization is scarcely developed in nonhuman primates.

## ACKNOWLEDGMENTS

We are grateful to H. Chen for her significant contribution to the implementation of pre- and postprocessing pipelines as well as MRI scanning; K. Willmes for his helpful advice on statistical measures; A. Schüppen and O. Poznansky for their notable inputs on DW-MRI processing techniques; J. Schreiber for his contribution to the ROI registration algorithm; A. Pellicano and H. Patel for their vital input regarding conceptualization. All figures in this study were conceptualized and illustrated by M.J.

## SUPPORTING INFORMATION

Supporting information for this article is available at https://doi.org/10.1162/netn_a_00407.

## AUTHOR CONTRIBUTIONS

Marvin Jüchtern: Data curation; Formal analysis; Investigation; Methodology; Software; Validation; Visualization; Writing – original draft; Writing – review & editing. Usman Jawed Shaikh: Formal analysis; Investigation; Methodology; Software; Validation. Svenja Caspers: Conceptualization; Formal analysis; Funding acquisition; Methodology; Project administration; Supervision; Writing – review & editing. Ferdinand Binkofski: Conceptualization; Formal analysis; Investigation; Methodology; Project administration; Resources; Supervision; Writing – review & editing.

## FUNDING INFORMATION

Svenja Caspers, Horizon 2020 Framework Programme (https://doi.org/10.3030/945539), Award ID: Grant Agreement No. 945539; HBP SGA3.

## DATA AVAILABILITY STATEMENT

The datasets generated and analyzed during the current study were newly acquired. All codes for pre- and postprocessing of this data were obtained from the software cited in the respective paragraph of the Materials and Methods section. Due to data privacy regulations of the study protocol by the ethics commission and national policy, raw data beyond the shared are available from the corresponding author on reasonable request with the need for a formal data sharing agreement. Anonymized raw connectivity matrices, aggregated whole-group bilateral thresholded connectivity, distance, node betweenness matrices, edge betweenness, and strength data are available via an open repository.

## Supplementary Material



## TECHNICAL TERMS

Visuomotor transformation: The ability of the brain to use visual information from the environment for steering object interaction like reaching.
Probabilistic tractography: Method of fiber-tracking between regions of interest, where every connection is computed multiple times to improve certainty of a tract.
Graph Theory Analysis: Set of mathematical tools used to analyze (brain) networks, abstracted into matrices of nodes (areas) and edges (links).
Multishell diffusion MRI: Combining different b-values (gradient strengths) in diffusion magnetic resonance imaging acquisition to enhance the distinction of brain tissues.
ROI parcellation: Division of the brain into distinct areas of interest, using microanatomical parameters to define borders.
Small-worldness: Feature of a network, having most of its components densely linked to each other with primarily short paths between them.
Distance matrix: Algorithm where strong connection between two areas is defined as fewer steps between them.
Betweenness centrality: Fraction of shortest paths that pass through a certain area (node) or tract (edge), serving high information flow.
Network hub: Brain area with many (local or distant) interconnections, collecting multiple pathways as a crossing point in a network.
