# Supplementary material for: A gradient of hemisphere-specific dorsal to ventral processing routes in parieto-premotor networks
Source: Netw Neurosci. 2024 Dec 10;8(4):1563–89. doi: 10.1162/netn_a_00407 (PMC11675101; doi:10.1162/netn_a_00407)
Supplement: Supplementary file 1 [file netn-8-4-1563-s001.pdf]

A gradient of hemisphere-specific dorsal to ventral processing routes in parieto-premotor networks

Marvin Jüchtern, Usman Jawed Shaikh, Svenja Caspers, Ferdinand Binkofski*

*Corresponding author

Supplementary Information

Supplementary Table S1: Centers of gravity for bilateral ROIs.

ROI	Region	Center of gravity (left)			Center of gravity (right)		
		**x**	**y**	**z**	**x**	**y**	**z**
**area 44**	Broca's area	-53.9794	11.8919	15.8690	53.0104	13.9334	11.1451
**area 45**	Broca's area	-54.8292	25.9638	13.4809	52.7346	26.2110	11.8623
**IFJ 1**	IFS	-39.6436	12.7633	27.8005	41.6804	14.3124	27.5012
**IFJ 2**	IFS	-42.0537	6.6205	29.1802	42.9124	10.0532	24.6078
**IFS 1**	IFS	-43.2171	25.7628	20.4729	36.8251	24.6539	25.2350
**IFS 2**	IFS	-47.2086	22.3669	19.4604	43.1404	22.5277	22.9787
**IFS 3**	IFS	-44.1136	19.3002	26.6592	38.4371	20.8099	31.6392
**IFS 4**	IFS	-45.4390	17.6411	23.4669	40.9650	18.7443	27.4495
**PM6d1**	Dorsal PMC	-20.5829	-12.9970	67.7510	17.7312	-13.7170	66.9080
**PM6d2**	Dorsal PMC	-15.9229	2.6091	67.0292	13.7112	4.7463	66.9976
**PM6d3**	Dorsal PMC	-24.8463	3.3126	51.7611	23.3651	3.1564	53.1656
**PMv**	Ventral PMC	-45.9165	-5.6425	42.3417	42.3408	-5.7022	43.9756
**PreSMA**	SMA	-6.2688	4.8571	59.0431	5.8505	5.5577	58.4333

**SMAproper**	SMA	-5.5723	-12.6094	58.2371	4.9288	-12.2091	57.1722
**hIP1**	AIPS	-38.7398	-50.4816	40.4940	40.0942	-48.1201	39.5121
**hIP2**	AIPS	-48.8519	-43.1190	43.8977	43.7025	-39.5734	45.3311
**hIP3**	AIPS	-33.8203	-54.7573	47.0963	33.6354	-50.0588	50.4098
**PF**	IPL	-60.9448	-39.8221	38.2931	63.4021	-32.5079	31.1655
**PFcm**	IPL	-54.4001	-39.1824	21.3720	53.1571	-30.4496	22.7693
**PFm**	IPL	-56.7237	-53.8279	39.1000	56.1549	-44.8225	39.9883
**PFop**	IPL	-62.4382	-26.3712	24.2890	57.4360	-18.9226	26.5730
**PFt**	IPL	-57.6409	-28.5513	38.0329	54.8611	-23.9959	40.1477
**PGa**	IPL	-49.5015	-63.6358	38.3259	54.4814	-55.6165	31.9179
**PGp**	IPL	-45.5082	-76.0439	29.6052	46.5857	-70.8689	31.3929
**hIP4**	PIPS	-31.5427	-82.0294	25.6175	36.0901	-77.7028	22.8209
**hIP5**	PIPS	-29.7859	-75.9316	37.4275	33.5763	-70.3133	34.2219
**hIP6**	PIPS	-32.4952	-68.5738	46.6840	36.7502	-63.7758	45.5222
**hIP7**	PIPS	-25.1072	-81.1633	28.0876	29.0901	-79.4012	27.8925
**hIP8**	PIPS	-19.4792	-74.0488	42.8054	25.4027	-72.7178	41.1179
**hPO1**	PIPS	-16.8912	-81.8600	36.1170	20.9806	-82.3062	36.9045
**5Ci**	SPL	-15.5055	-35.5720	42.6495	10.3951	-35.6529	45.4372
**5L**	SPL	-19.1724	-45.8544	67.2100	14.4691	-48.8223	68.9292
**5M**	SPL	-9.1715	-40.4434	56.5064	5.8387	-43.3059	58.2823
**7A**	SPL	-21.6067	-61.8777	61.1651	21.6355	-62.1126	61.4568
**7M**	SPL	-3.2972	-78.6050	35.2193	3.4521	-76.3243	36.1155
**7P**	SPL	-9.0452	-74.1343	52.7977	14.9366	-74.5280	54.6909
**7PC**	SPL	-34.8194	-48.7253	62.2392	29.9359	-47.5146	62.1250

10

Supplementary Table S2: Voxel-Size and corresponding cortical areas in primates for the network's ROIs.

ROI	Region	size (left)	size (right)	Putative correspondance in macaques
**area 44**	Broca's area	5252	4233	area 44[1]or ProM[2]
**area 45**	Broca's area	3477	5934	area 45B[1] or area 45(A & B)[2]
**IFJ 1**	IFS	376	413	area 44[2]
**IFJ 2**	IFS	838	696	area 44[2]
**IFS 1**	IFS	645	549	area 45B[2]
**IFS 2**	IFS	139	235	area 45B[2]
**IFS 3**	IFS	493	826	area 45B[2]
**IFS 4**	IFS	287	485	area 45B[2]
**PM6d1**	Dorsal PMC	5895	6421	F2 & F7[3]
**PM6d2**	Dorsal PMC	4070	4158	F2 & F7[3]
**PM6d3**	Dorsal PMC	3122	4500	F2 & F7[3]
**PMv**	Ventral PMC	6767	6223	F4 & F5[3]
**PreSMA**	SMA	3065	2315	F6[3]
**SMAproper**	SMA	1915	1980	F3[3]
**hIP1**	AIPS	3482	2781	AIP[4] or AIP/VIP[5]
**hIP2**	AIPS	2404	1758	AIP[6] or AIP/VIP[5]
**hIP3**	AIPS	3605	3843	AIP[7] or MIP[4,6]
**PF**	IPL	4565	6253	PFG[8,9]
**PFcm**	IPL	3382	2527	PF[9]
**PFm**	IPL	5352	6748	PFG[9]
**PFop**	IPL	2104	1782	PF[8,9]

**PFt**	IPL	4442	3427	AIP[5,6] or PF[8,9]
**PGa**	IPL	5444	6616	PG[8,9]
**PGp**	IPL	6405	8399	PG[9] or Opt[8,10]
**hIP4**	PIPS	1498	1820	CIP[11]
**hIP5**	PIPS	3027	2091	LIP/CIP[11]
**hIP6**	PIPS	2886	2698	LIP/CIP[11]
**hIP7**	PIPS	1427	2475	PIP[11]
**hIP8**	PIPS	2502	2332	PIP[11]
**hPO1**	PIPS	2565	2681	V2/3/6[11]
**5Ci**	SPL	1007	1354	PEci[4,12]
**5L**	SPL	4548	4125	PE[4,12]
**5M**	SPL	3049	1860	PE[4,12]
**7A**	SPL	9163	6055	LIP(ant)[6,7,13,14]
**7M**	SPL	757	407	7m[4]
**7PC**	SPL	2370	3809	VIP[13,15] or *none*[12]
**7P**	SPL	2852	3267	PEc[4]

Supplementary Figure S1: Topography of left-sided fronto-parietal networks.

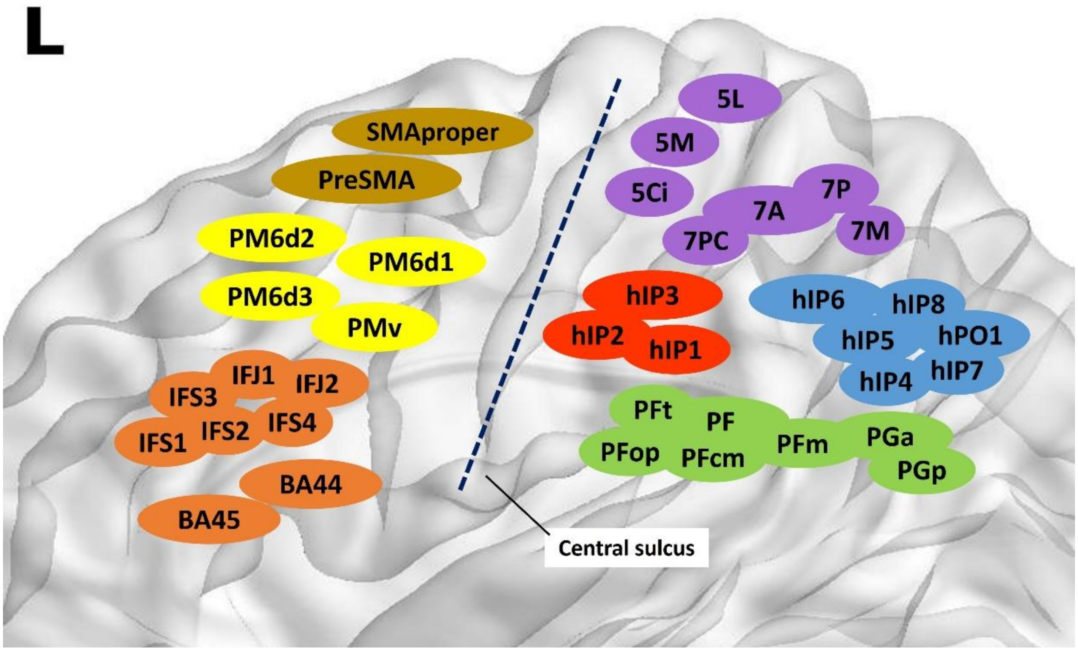

ROIs are depicted as single cortical areas in colored spheres. Coloring refers to corresponding anatomical regions: Ventrolateral prefrontal cortex (orange), premotor cortex (yellow), supplementary motor area (brown), inferior parietal lobule (green), anterior intraparietal sulcus (red), posterior intraparietal sulcus (blue), superior parietal lobule (purple). *Area 44/area 45 = Broca's area; IFS = inferior frontal sulcus; IFJ = inferior frontal junction; PMv = ventral premotor cortex; PMd = dorsal premotor cortex; SMA = supplementary motor area; area 5 = anterior SPL; area 7 = posterior SPL; PF = supramarginal gyrus; PG = angular gyrus; hIP1-3 = human anterior intraparietal sulcus (aIPS); hIP4-8/hPO1 = human posterior intraparietal sulcus (pIPS).*

Supplementary Figure S2.1: Coefficients of variation in the left hemisphere.

LEFT	area 44	area 45	IFJ1	IFJ2	IFS1	IFS2	IFS3	IFS4	PM6d1	PM6d2	PM6d3	PMv	PreSMA	SMA
hIP1	1,15580845	1,68854714	1,57086112	1,60139177	1,51088671	1,37702568	1,98064495	1,60357917	1,11126487	1,44363276	1,14019311	0,71881632	0,67527418	0,84183079
hIP2	0,68157784	1,24612863	1,80951497	1,19236285	1,20398061	1,35343817	1,1019488	1,24239076	0,96184356	0,77497257	1,42800104	0,67877312	0,81030416	0,80859701
hIP3	1,0176298	1,01573315	2,02763298	1,76573748	2,03363923	1,46176337	2,26192824	1,5688061	1,02128469	1,19383059	0,98411368	0,84729132	0,8268529	3,12598082
PF	0,51994971	0,69781401	1,86569043	0,89494395	0,8846373	0,96982807	0,76994613	0,91067766	1,06579033	0,67448272	1,08691385	0,79101156	0,74318141	1,03577252
PFcm	0,64380472	0,93530108	2,82567672	1,64080417	1,54235095	1,86218663	1,29426387	1,39233998	1,21570194	0,68043833	0,89830431	2,16376227	0,85460546	1,37742574
PFm	0,81316284	1,35882529	1,30829583	1,21849226	1,35250756	1,39445985	1,67978173	1,3923038	1,07410858	0,69432928	1,52584026	0,82156023	0,63845819	0,99974262
PFop	0,57976316	0,73486083	2,12617665	1,39209746	1,00959583	2,07132641	1,47461153	1,63020538	2,06515603	0,93530749	0,72944431	1,31239452	1,17593434	1,02180587
PFt	0,51180855	0,76708218	1,5820938	1,1241552	0,80060061	1,56164863	0,82035351	1,33796846	1,6995452	0,81371182	0,86439245	0,84082125	0,87354138	1,59514258
PGa	0,87146283	1,32121513	1,48232251	1,25873277	1,80693013	1,73822088	1,71454044	1,64029136	0,98965129	1,20260768	1,59665311	0,81776344	0,7337658	0,92655249
PGp	0,95350279	1,02880505	1,39318435	1,1707106	1,7581785	1,68958917	1,35806363	1,33556932	0,92293423	0,87988458	1,43963408	1,09930628	0,92498118	0,9259283
hIP4	0,97406213	1,15927367	1,98427711	1,8435544	1,14060013	1,65720987	1,25891513	2,5540315	1,00941413	1,20525703	1,51845006	1,7206341	1,01537497	0,92789024
hIP5	1,02775593	1,39398834	1,33100029	1,71746814	1,31185275	1,77782138	1,20281423	3,08026231	0,78245083	0,88750688	1,27537919	1,27062394	1,086423	0,93721347
hIP6	0,97998692	0,91020223	1,86899787	1,48817763	1,90502562	1,70021715	1,80701117	1,73201714	0,79903801	0,98921146	0,87076733	1,25967368	0,73707677	1,24851413
hIP7	1,05938204	0,97829524	2,71098061	1,75888267	1,10360714	1,89386091	1,4459498	2,36461368	1,18323735	1,28951797	1,90180144	0,92118349	1,17257055	1,33217911
hIP8	0,80217269	0,98561268	1,28990895	1,80347675	0,96594432	1,0505796	0,85156613	1,55739405	1,36840112	1,06863428	1,81849671	1,75647485	1,00091724	1,98437332
PO1	0,75229372	0,82999872	1,27282921	1,43857176	1,11113479	1,01273937	0,89629708	1,19984107	1,4370928	1,24465774	1,72526178	0,99672714	1,06938351	2,4666034
5Ci	0,95585502	1,02146053	1,23839189	1,25556949	1,1278537	1,52068691	1,10008128	1,23308451	0,99537257	1,08991473	1,4989315	1,00520855	0,64713367	0,52405496
5L	0,57778479	0,66885527	1,09896697	1,19701603	1,03487375	1,13206297	0,84088397	1,34149921	0,7276565	0,75728508	0,79132096	0,78831365	0,64017928	0,76900859
5M	0,73996451	0,85235264	1,04027172	0,95413963	1,14529654	1,35885415	1,06680527	1,13410832	0,69294418	0,95593168	1,63032853	0,88348509	0,63547413	0,70226168
7A	0,57442718	0,71496825	1,09168582	1,18145793	1,06082005	0,99042621	1,04359127	0,86405621	0,69067764	0,71599789	0,78460621	0,89287535	0,63803468	0,94016906
7M	0,72142169	0,8497685	1,14020857	1,31319108	1,1769658	1,15962466	1,00859727	0,99036151	0,88289559	1,03085005	1,40529879	0,81059301	0,82920693	0,95486792
7PC	0,82289525	0,71170663	1,26742419	1,76451033	0,91185896	0,80987886	1,28527396	1,13094493	0,81891931	0,90521466	0,81334591	0,81446478	0,76807719	1,20302198
7P	0,71648737	0,89213166	1,03096263	1,52516459	1,31717621	1,43265342	1,10787037	0,97482707	0,99596704	1,01013614	1,61467058	0,82913128	0,81634713	0,91099657

< 1st Quartile	< 2nd Quartile	< 3rd Quartile	≥ 3rd Quartile

Coefficient of variation (CV) values of left-sided ROI-to-ROI connectivity paired-sample t-tests across forty subjects. Cells in white contain CV values below the first quartile of all CV values (< 0.8720, Cluster A). Cells in orange contain CV values below the second quartile of all CV values (< 1.0908, Cluster B). Cells in yellow contain CV values below the third quartile of all CV values (< 1.3943, Cluster C). Cells in white contain CV greater or equal the third quartile of all CV values (Cluster D).

Supplementary Figure S2.2: Coefficients of variation in the right hemisphere.

RIGHT	area 44	area 45	IFJ1	IFJ2	IFS1	IFS2	IFS3	IFS4	PM6d1	PM6d2	PM6d3	PMv	PreSMA	SMA
hIP1	0,76875391	1,00738495	1,01035315	1,21400569	1,1851772	1,28975687	1,31485381	1,39134996	0,96585969	0,77378582	1,53791163	0,79746394	0,64683905	0,81983663
hIP2	0,65108434	1,00391178	1,03401163	1,10184597	1,60955256	1,31514601	1,49129725	1,20365094	0,98537345	0,85195659	1,60865091	0,76215879	0,7748921	0,83250604
hIP3	0,88365028	0,93295935	1,23287429	1,29257375	1,25172181	1,18326328	1,28284141	1,41785635	0,95814734	0,80311365	1,20375461	0,59007575	0,6021675	1,09361053
PF	0,61601554	0,75903198	1,13395434	1,42960128	0,93472753	1,83899105	1,02713523	1,26672794	0,98760305	0,53601711	0,63134464	0,80993431	0,62113625	0,77076853
PFcm	0,972565	0,74641611	3,91551601	3,19209669	3,94999635	2,97212665	3,72444038	3,73179056	0,84590377	0,55044525	0,63300499	0,75904732	0,68423869	0,80511187
PFm	0,61708922	0,72868035	0,84560928	0,85066752	0,99015095	1,48264909	1,02185227	1,14765726	0,68649054	0,62089105	1,37765819	0,72463967	0,64733711	0,94949386
PFop	0,80827397	0,79587405	0,88245535	1,82167497	0,9877018	1,36831724	0,90477883	0,99047864	1,42159872	0,66209773	0,77391553	1,13710634	0,93992074	1,12142378
PFt	0,49546107	0,67079717	0,91533622	1,24851399	1,16532957	0,99071744	1,37270947	1,11932409	1,84020464	0,7213843	0,8842501	1,06214764	0,64572078	0,92498935
PGa	1,11313199	0,84926415	0,92423261	0,93340557	1,02367029	1,24316226	1,09974364	1,17896528	0,61706074	0,71985771	1,9558106	0,88503937	0,76443661	0,94408745
PGp	0,94910534	1,05659353	1,28077122	1,45514544	1,33358371	1,48766503	1,46960124	1,41244174	0,85058106	0,88153391	1,04836868	0,72232091	1,0695775	1,90997025
hIP4	1,2860943	1,03529032	1,96303904	2,39022403	1,68717491	2,07627008	1,74341839	1,72298026	1,10714322	1,10578317	1,16357185	2,16990161	1,79631023	2,35806476
hIP5	1,11870741	1,12051341	1,83487721	1,99443019	2,00606833	2,53318998	1,77899909	1,85194257	1,06388334	1,03038296	1,08990464	1,33515721	1,7729745	2,12434002
hIP6	1,26893366	1,25847745	1,85378962	2,37529588	1,65988422	2,15189901	1,8750384	2,25243928	0,9323441	0,99903228	1,27748473	0,89792953	0,85839846	0,84193995
hIP7	1,18442666	0,85823819	1,98984383	2,5445211	1,99940402	1,91725965	1,97176019	1,75187722	1,18600299	1,30015945	1,14772243	2,22589604	1,70307773	1,67727994
hIP8	0,87513387	0,66619697	2,31762368	1,87497453	2,18186698	2,89721352	1,87125058	2,38112234	1,0181652	1,04053441	1,25785829	1,96440676	1,23329048	1,393252
PO1	0,81582077	0,70948952	2,03759813	2,26283878	2,0058149	1,95441915	2,19859018	1,83340618	0,73354306	0,75109777	1,3299239	1,36051541	0,74872987	1,07578449
5Ci	1,02011643	1,09576684	1,30937106	2,38316747	0,88678084	0,99188365	0,91049945	1,16572219	1,23952567	0,74658102	1,67914554	0,9368385	0,65272886	0,7980477
5L	0,64330779	0,51241633	1,27469613	2,2255269	0,90990861	0,87955303	1,02628817	0,87945006	0,48391906	0,74124544	1,02719926	0,75209381	0,64291275	0,77551604
5M	0,80734019	0,65677756	1,35603426	2,61515157	0,89280205	1,12415682	0,86418066	0,998643	0,52119755	0,61862458	1,35422411	0,75249409	0,55831485	0,72849752
7A	0,6345526	0,55018923	1,34162041	1,57290511	1,14637312	1,27472638	1,32636773	1,81177998	0,64612186	0,60691513	0,95006952	0,81488184	0,79668782	1,12398412
7M	0,88375254	1,54207785	1,1196255	2,24179415	0,89176087	0,85693331	0,8478995	1,23177539	1,57086538	1,15335582	1,16338965	0,81885591	1,20967086	1,89080848
7PC	0,76219367	0,53451721	1,52430877	1,29826939	1,16961024	1,29421323	1,11370947	1,07169913	0,55271041	0,94985579	0,99376989	0,6734935	0,69115339	1,3170429
7P	0,8506329	0,72529012	1,02998583	1,0718694	0,91586789	1,31656478	0,86322628	1,28354877	1,04376265	0,78871578	1,3409723	0,80354539	0,79560031	1,3424366

< 1st Quartile < 2nd Quartile < 3rd Quartile ≥ 3rd Quartile

Coefficient of variation (CV) values of right-sided ROI-to-ROI connectivity paired-sample t-tests across forty subjects. Cells in white contain CV values below the first quartile of all CV values (< 0.8230, Cluster A). Cells in orange contain CV values below the second quartile of all CV values (< 1.0706, Cluster B). Cells in yellow contain CV values below the third quartile of all CV values (< 1.3879, Cluster C). Cells in white contain CV greater or equal the third quartile of all CV values (Cluster D).

Supplementary Figure S3: Schematic depiction of hub roles in the network.

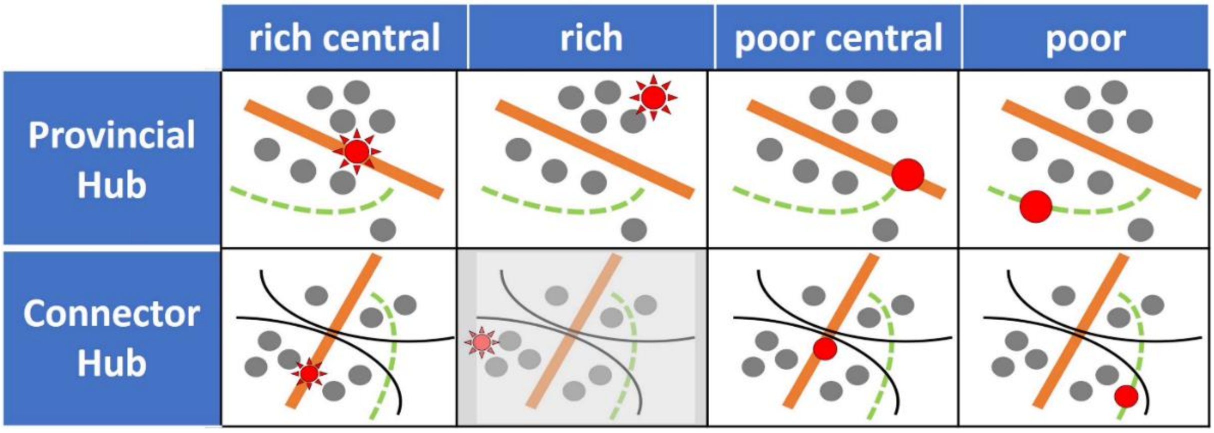

A red sphere symbolizes the given node; small arrow heads around it ("sun shape") reflect strong local linkage; gray spheres stand for neighboring nodes. Black lines mark module-boundaries, while thick orange and dotted green lines represent larger and smaller fiber tracks, respectively. Note that there were no rich connector hubs.

Supplementary Methods: Code for connectedness calculation in matlab®.

```
g = digraph(A);
bins = conncomp(g, 'Type', 'weak');
isConnected = all(bins == 1);
```

For "A" as the respective matrix and "isConnected" = 1 proving connectedness of the graph.

*Reference: Christine Tobler, 2019, https://de.mathworks.com/matlabcentral/answers/39698-connected-graph-given-adjacency-matrix (*last access on the 3rd of June 2024).

Supplementary Discussion: Node character assignment approach.

In the current study we propose a refined approach for node characterization through a two-step evaluation process, inspired by the works of Guimerà and Amaral as well as subsequent modifications of this approach[16,17]. The original strategy also comprised two stages of node validation, with the first being hub-identification via the within-module degree z-score (WMDZ) and the second focusing on hub types, using the participation coefficient (PC)[18]. It was later argued that such analyses were too much dependent on network and node size, reasoning that not only the quantity, but also the quality of connections should be considered for hub characterization and that a hodological approach could be preferable for structural graphs[19]. We tried to take these arguments into consideration, using quartiles rather than fixed cut-off values and including node-betweenness centrality (NBC) as an attempt to qualitatively accentuate node strength by a shortest-path-based connectivity measure, similarly described by Sporns et al.[20].

In the current approach, WMDZ and PC defined provincial and connector hubs, respectively, deeming either function specific for determination of one of the two hub types. While provincial hubs usually facilitate modular segregation, connector hubs appear to be important for global intermodular integration[21]. Hubs classified as “rich central” are putatively serving as waypoints on highly frequented network routes, either on a local or (supra)regional scale. Interestingly, we did not find “rich” connector hubs, consistent with the assumption that “rich” means locally well-intertwined but beyond larger long-distance pathways and therefore not congruous with the profile of connectors. On the other hand, we associate “poor central” with hubs located nearby passages of important traffic routes, getting relatively low input from neighboring nodes, while “poor” provincial and connector hubs recall positions on rather secondary network paths, similarly lacking strong integration into local connectivity. It is debatable whether the latter (two in the current study) can be viewed as actual hubs, resembling features of non-hubs, and asking for future validation.

Peripheral nodes on the other hand showed lowest scores for strength and NBC alike, making these presumably far more secluded and less interacting with other domains in the network. Importantly, a hub might be classified as a fronto-parietal connector hub even if it appears less central or even less coupled than surrounding areas. Apart from statistical reasons, this is mainly due to the fact, that the algorithm doesn't differ between inter- and intralobular clusters. Thus, a hub might link several modules around it in a central manner, without having the same role for long distances, i.e., for fronto-parietal pathways. Since we only focused on the latter in interpretation of the results, such divergences can happen without disproving the stability of the approach.

Supplementary References

1 Frey, S., Mackey, S. & Petrides, M. Cortico-cortical connections of areas 44 and 45B in the macaque monkey. *Brain Lang* **131**, 36-55, doi:10.1016/j.bandl.2013.05.005 (2014).

2 Neubert, F. X., Mars, R. B., Thomas, A. G., Sallet, J. & Rushworth, M. F. Comparison of human ventral frontal cortex areas for cognitive control and language with areas in monkey frontal cortex. *Neuron* **81**, 700-713, doi:10.1016/j.neuron.2013.11.012 (2014).

3 Geyer, S., Matelli, M., Luppino, G. & Zilles, K. Functional neuroanatomy of the primate isocortical motor system. *Anat Embryol (Berl)* **202**, 443-474, doi:10.1007/s004290000127 (2000).

4 Scheperjans, F. *et al.* Observer-independent cytoarchitectonic mapping of the human superior parietal cortex. *Cereb Cortex* **18**, 846-867, doi:10.1093/cercor/bhm116 (2008).

5 Choi, H. J. *et al.* Cytoarchitectonic identification and probabilistic mapping of two distinct areas within the anterior ventral bank of the human intraparietal sulcus. *J Comp Neurol* **495**, 53-69, doi:10.1002/cne.20849 (2006).

6 Mars, R. B. *et al.* Diffusion-weighted imaging tractography-based parcellation of the human parietal cortex and comparison with human and macaque resting-state functional connectivity. *J Neurosci* **31**, 4087-4100, doi:10.1523/JNEUROSCI.5102-10.2011 (2011).

7 Durand, J. B., Peeters, R., Norman, J. F., Todd, J. T. & Orban, G. A. Parietal regions processing visual 3D shape extracted from disparity. *Neuroimage* **46**, 1114-1126, doi:10.1016/j.neuroimage.2009.03.023 (2009).

8 Caspers, S. *et al.* Probabilistic fibre tract analysis of cytoarchitectonically defined human inferior parietal lobule areas reveals similarities to macaques. *Neuroimage* **58**, 362-380, doi:10.1016/j.neuroimage.2011.06.027 (2011).

9 Ruschel, M. *et al.* Connectivity architecture and subdivision of the human inferior parietal cortex revealed by diffusion MRI. *Cereb Cortex* **24**, 2436-2448, doi:10.1093/cercor/bht098 (2014).

10 Averbeck, B. B., Battaglia-Mayer, A., Guglielmo, C. & Caminiti, R. Statistical analysis of parieto-frontal cognitive-motor networks. *J Neurophysiol* **102**, 1911-1920, doi:10.1152/jn.00519.2009 (2009).

11 Richter, M. *et al.* Cytoarchitectonic segregation of human posterior intraparietal and adjacent parieto-occipital sulcus and its relation to visuomotor and cognitive functions. *Cereb Cortex* **29**, 1305-1327, doi:10.1093/cercor/bhy245 (2019).

12 Scheperjans, F. *et al.* Probabilistic maps, morphometry, and variability of cytoarchitectonic areas in the human superior parietal cortex. *Cereb Cortex* **18**, 2141-2157, doi:10.1093/cercor/bhm241 (2008).

13 Orban, G. A. *et al.* Mapping the parietal cortex of human and non-human primates. *Neuropsychologia* **44**, 2647-2667, doi:10.1016/j.neuropsychologia.2005.11.001 (2006).

14 Vanduffel, W., Zhu, Q. & Orban, G. A. Monkey cortex through fMRI glasses. *Neuron* **83**, 533-550, doi:10.1016/j.neuron.2014.07.015 (2014).

15 Sereno, M. I. & Huang, R. S. A human parietal face area contains aligned head-centered visual and tactile maps. *Nat Neurosci* **9**, 1337-1343, doi:10.1038/nn1777 (2006).

16 Meunier, D., Lambiotte, R., Fornito, A., Ersche, K. D. & Bullmore, E. T. Hierarchical modularity in human brain functional networks. *Front Neuroinform* **3**, 37, doi:10.3389/neuro.11.037.2009 (2009).

17 Cohen, J. R. & D'Esposito, M. The Segregation and Integration of Distinct Brain Networks and Their Relationship to Cognition. *J Neurosci* **36**, 12083-12094, doi:10.1523/JNEUROSCI.2965-15.2016 (2016).

18 Guimera, R. & Nunes Amaral, L. A. Functional cartography of complex metabolic networks. *Nature* **433**, 895-900, doi:10.1038/nature03288 (2005).

19 Power, J. D., Schlaggar, B. L., Lessov-Schlaggar, C. N. & Petersen, S. E. Evidence for hubs in human functional brain networks. *Neuron* **79**, 798-813, doi:10.1016/j.neuron.2013.07.035 (2013).

20 Sporns, O., Honey, C. J. & Kotter, R. Identification and classification of hubs in brain networks. *PLoS One* **2**, e1049, doi:10.1371/journal.pone.0001049 (2007).

21 Rubinov, M. & Sporns, O. Complex network measures of brain connectivity: uses and interpretations. *Neuroimage* **52**, 1059-1069, doi:10.1016/j.neuroimage.2009.10.003 (2010).
